# Psychological Well-Being of Mothers of Children With Autism in Saudi Arabia

**DOI:** 10.7759/cureus.23284

**Published:** 2022-03-18

**Authors:** Khaled Alghamdi, Shahad Alahmadi, Abeer Sayedahmad, Hanan Mosleh

**Affiliations:** 1 Pediatrics, Taibah University, Madinah, SAU; 2 Preventive Medicine, Ministry of Health Holdings, Madinah, SAU; 3 Special Education, Ministry of Education, Madinah, SAU; 4 Department of Family and Community Medicine, College of Medicine, Taibah University, Madinah, SAU; 5 Public Health and Community Medicine Department, Cairo University, Cairo, EGY

**Keywords:** psychological well-being, autism, stress, anxiety, depression

## Abstract

Introduction

Previous studies reported that parents of children with autism spectrum disorder (ASD) experience higher levels of stress than parents of children with typical development.

Methods

This study conducted a quantitative cross-sectional survey to evaluate the levels of depression, anxiety, and stress among mothers of children with ASD compared with mothers of children with typical development. In addition, we assessed whether the perception of social support is correlated to psychological well-being. The study recruited a non-probability sample of 143 mothers of children with ASD and a comparison group of 143 mothers of children with typical development. For data collection, an online questionnaire was used to investigate the levels of depression, anxiety, and stress using the 21-item Depression Anxiety Stress Scale (DASS-21) and examine social support for mothers using the Multidimensional Scale of Perceived Social Support (MSPSS).

Results

No significant difference was observed in the levels of depression and anxiety between both groups. Nevertheless, extremely severe levels of depression and anxiety were observed in 23.1% and 27.3% of mothers in the ASD group compared with 11.9% and 16.8% for the control group, respectively. Alternatively, a significant difference exists between the two groups in the levels of stress. Extremely severe stress was identified in 17.5% of mothers of children with ASD compared with only 6.3% in the control group (p = 0.04). Lastly, the study found a significant correlation among the scores for stress, anxiety, and depression and for MSPSS.

Conclusion

Providing mental health services for mothers in need and investigating the underlying factors of extremely severe levels of depression and anxiety are recommended initiatives.

## Introduction

Autism spectrum disorder (ASD) is a neurodevelopmental disorder with an early onset and manifests as unusual behaviors, such as negligence, independently undertaking tasks, isolationism, and constant repetitive tasks. This tendency leads to a disturbance in interpersonal emotional communication and failure to develop peer relationships. It is characterized by language delay in the majority of patients and other mental health issues [1].

According to a systematic review published in 2014, the prevalence of ASD in Arab Gulf countries is 1.4-29 per 10,000 individuals. Numerous risk factors have been identified for the development of such conditions, including metabolic, autoimmune, and environmental factors. Moreover, perinatal complications, cesarean section, suboptimal breastfeeding, and parent age were considered to have potential associations with ASD [2].

Providing full services to individuals with ASD and their families is a challenging task. Despite improvement in this regard, Saudi Arabia is similar to other countries and faces various barriers, such as misdiagnosis, late diagnosis, lack of population awareness, and limited availability of services [3].

Scholars reported that parents of children with ASD experience higher levels of stress, depression, and anxiety than parents of children with normal development [4]. In China, 72.5% of mothers caring for children with ASD developed depressive symptoms, 80.2% developed anxiety, and 67.1% suffered from both symptoms of the illness [5-7]. Several studies from our region such as Bahrain and Oman and other countries reported different manifestations of negative psychological impacts on caregivers of children with ASD [8-12]. However, studies that focused on the correlation of such an impact with the perceived social support by mothers of children with ASD are lacking.

To fill this research gap, this study aimed to evaluate the levels of depression, anxiety, and stress among mothers of children with ASD compared with mothers of children with normal development. Moreover, we examined the social support received by mothers of children with ASD and whether their psychological well-being is correlated with the perception of social support.

## Materials and methods

Study design

This study is cross-sectional in nature.

Study settings and study period

The study recruited a non-probability convenient sample of mothers of children with ASD (N = 143) who joined autism support centers in Madinah and Riyadh in Saudi Arabia. This group was matched with a control group of mothers of children with normal development (N = 143). The study was conducted from June 1, 2020, to September 2020.

Study population and sampling

The sample size was calculated using the Open Epi software based on a prevalence of 1.4-29 per 10,000 persons, such that a size of between 22 and 307 is acceptable. An electronic version of the questionnaire was disseminated to different special schools for children with ASD in Madinah and Riyadh. Volunteering mothers were recruited through principals who circulated the questionnaire using their database.

Data collection

The questionnaire consisted of four parts. The first part included sociodemographic details (e.g., age of the mother; age of the child; work status of the mother, if any; and the monthly income of the family).

The second part was intended for the mothers of children with ASD only and included the obstetric history of the mother at birth of the child with ASD, such as the age of both parents at birth, type of delivery (normal vaginal delivery or cesarean section), whether the baby was full term, and diseases associated with the pregnancy.

The third part consists of the Arabic version of the 21-item Depression Anxiety Stress Scale (DASS-21), which is proven to have acceptable reliability [13]. It contains three subscales, namely, depression, anxiety, and stress, with seven items per subscale. Items were rated using a scale from 0 (never) to 3 (almost always). The scores are summed by adding up the scores per subscale and multiplying them by a factor of two to be equivalent to the longer DASS-42 version. Thus, each subscale may range between 0 and 42 [14]. The sum of responses to items 3, 5, 10, 13, 16, 17, and 21 denotes the score for the depression subscale, whereas those for items 2, 4, 7, 9, 15, 19, and 20 represent the anxiety subscale. Lastly, items 1, 6, 8, 11, 12, 14, and 18 would give the score for the stress subscale.

The fourth part was also intended for mothers of children with ASD only and consists of the Arabic version of the Multidimensional Scale of Perceived Social Support (MSPSS) [15]. It is a short 12-item questionnaire that measures the perceived social support of mothers derived from three main sources, namely, family, friends, and significant others. Items are rated using a seven-point Likert-type scale ranging from 1 (very strongly agree) to 7 (very strongly disagree) with a maximum score of 84. The higher the MSPSS score, the higher the perceived social support. The internal consistency of the MSPSS-Arabic version is high (Cronbach’s alpha = 0.87), which is a value comparable to the English version of the MSPSS [15].

Statistical analysis

Data were entered on Excel (Microsoft Corp., Redmond, WA, USA), whereas analysis was conducted using SPSS version 21 (IBM Corp., Armonk, NY, USA). Descriptive statistics were performed using the frequencies and percentages of the qualitative variables. For the quantitative variables, measures of central tendency (median) and dispersion (minimum and maximum) were used, because they were non-normally distributed when examined using the Shapiro-Wilk test. The levels of depression, anxiety, and stress between mothers of children with ASD and mothers of children with typical development were compared using the Chi-squared test. Spearman’s rho correlation was used to examine the correlation of the domains of the DASS scores (i.e., stress, anxiety, and depression) with the MSPSS score (perceived social support) among mothers of children with ASD. Statistical significance was set at p < 0.05.

Ethical considerations

Ethical approval was obtained from the ethical committee of the College of Medicine, Taibah University (study ID 019-1442 on October 26, 2020; IRB 00010413). Furthermore, the participants provided informed consent prior to the study. All procedures conformed to the ethical principles of the Declaration of Helsinki for medical research involving human subjects [16].

## Results

Demographic data and risk factors for autism (ASD group)

Among mothers in the ASD group, 76.9% were less than 40 years old. However, 61.5% were not working outside the home (housewives), whereas 60.1% generated a monthly income of less than 15,000 Saudi Riyal (SR). Table 1 presents the important demographic data and perinatal history of children with ASD.

**Table 1:** Demographic data of the two groups and risk factors associated with autism in the ASD group

	Mothers of children with ASD (N = 143 (%))	Mothers of children with typical development (N = 143 (%))	
Age (years)			
20–39	110 (76.9%)	90 (62.9%)	
40 and above	33 (23.1%)	53 (37.1%)	
Work status			
Non-working outside the home	88 (61.5%)	78 (54.5%)	
Currently working	55 (38.5%)	65 (45.5%)	
Monthly income			
Less than 15,000	86 (60.1%)	88 (61.5%)	
15,000 and above	57 (39.9%)	55 (38.5%)	
Age of mother at birth of a child with ASD (in years) (N = 143 (%))			
<20			8 (5.6%)
20–29			82 (57.3%)
30–39			43 (30.1%)
40–49			10 (7%)
Mode of delivery			
Normal vaginal delivery			89 (62.2%)
Cesarean section			54 (37.8%)
Gestational age			
Full term			103 (72%)
Preterm			40 (28%)
Diseases associated with pregnancy			
Yes			32 (22.4%)
No			111 (77.6%)

Source of social support

With respect to the social support offered to mothers of children with ASD, 30.1% mentioned the spouse as the most supportive person, followed by others at 21%, brother/sister or parents at 16% for each, and close friends at 13%. The median for the MSPSS score was 50.41, where scores between 12 and 84 indicate a moderate level of social support.

Levels of depression, anxiety, and stress

For depression, 23.1% of mothers of children with ASD suffered from extremely severe depression compared with 11.9% of mothers of children with normal development (p = 0.055). In addition, 27.3% of mothers of children with ASD and 16.8% of mothers of children with normal development suffer from extremely severe levels of anxiety with no significant difference between the two groups (p = 0.235). However, the level of stress among mothers of children with ASD was statistically significantly different from that of mothers with children with normal development (p = 0.042). Moreover, 17.5% of mothers of children with ASD suffered from extreme stress compared with 6.3% of mothers of children with typical development. Table 2 provides an exact report of the levels of depression, anxiety, and stress among mothers of children with ASD and mothers of children with normal development at different levels.

**Table 2:** Levels of depression, anxiety, and stress among mothers of children with ASD and mothers of children with typical development

	Mothers of children with ASD (N = 143 (%))	Mothers of children with normal development (N = 143 (%))	p
Levels of depression			
Normal	54 (37.8%)	62 (43.4%)	0.055
Mild	16 (11.2%)	18 (12.6%)	
Moderate	29 (20.3%)	34 (23.8%)	
Severe	11 (7.7%)	12 (8.4%)	
Extremely severe	33 (23.1%)	17 (11.9%)	
Levels of anxiety			
Normal	61 (42.7%)	63 (44.1%)	0.235
Mild	8 (5.6%)	9 (6.3%)	
Moderate	23 (16.1%)	29 (20.3%)	
Severe	12 (8.4%)	18 (12.6%)	
Extremely severe	39 (27.3%)	24 (16.8%)	
Levels of stress			
Normal	58 (40.6%)	69 (48.3%)	0.042
Mild	14 (9.8%)	20 (14%)	
Moderate	18 (12.6%)	15 (10.5%)	
Severe	28 (19.6%)	30 (21%)	
Extremely severe	25 (17.5%)	9 (6.3%)	

Perceived social support and psychological well-being of mothers of children with ASD

The analysis demonstrated an inverse, weak-to-moderate, and significant correlation among the scores for stress, anxiety, and depression and the MSPSS. As illustrated in Table 3, the higher the level of perceived social support (MSPSS score), the better the psychological well-being of the mothers, particularly with respect to stress, anxiety, and depression (DASS scores).

**Table 3:** Correlation of scores for stress, anxiety, and depression with perceived social support *Statistically significant at p < 0.05

DASS scores	MSPSS score	
	Spearman’s correlation coefficient (r)	p
Stress	−0.365	0.0001*
Anxiety	−0.336	0.0001*
Depression	−0.330	0.0001*

## Discussion

The current study intended to explore the psychological well-being of mothers of children with ASD in comparison with mothers of children with normal development and whether it was correlated with perceived social support for the ASD group. Mothers in the ASD group were younger than the other group. However, their job “work outside the home” status was less than the comparison group. This finding is expected because mothers of children with ASD play the role of main caregivers [17]. Although both parents are prone to experience more distress [18], this behavior is common among mothers taking care of children with ASD. Thus, they tend to work inside the home, as reported by similar studies in Bahrain [8]. Nevertheless, both groups generated similar levels of monthly income.

The validated DASS-21 tool is commonly used to assess the psychological well-being of adults and has been previously utilized in Oman and Bahrain [8,9] for the same objective as that of the current study.

Stress is a major manifestation of stressful events in life, which was assessed over time beginning with the early diagnosis of ASD up to two years later among other determinants of psychological well-being, including anxiety and depression, in mothers of children with ASD in Bahrain. Depression and anxiety were determined to occur earlier; however, only stress is maintained [8]. Moreover, the study did not specify the time of diagnosis for the ASD group. However, with respect to stress, the results were comparable to those of previous studies, where the level of stress is higher for mothers of children with ASD, which was statically evident [8-10].

With respect to depression and anxiety, the current study found no significant difference between the two groups. Several studies were conducted to assess these manifestations. The present study demonstrated that 62.2% of the mothers of children with ASD experience varying degrees of depression, which range from mild to extremely severe, compared with 56.6% of mothers in the control group. This finding is comparable with those of other studies in our region that utilize the same instruments and settings. For example, Al Tourah et al. [8] and Al-Farsi et al. [9] conducted a study in Oman and Bahrain, respectively, and reported that 57% and 43% of mothers of children with ASD showed varying levels of depression. To reiterate, the current study found that 57.3% of mothers of children with ASD experienced certain levels of anxiety that range from mild to extremely severe compared with 55.9% in the control group. This finding is in agreement with those of other studies in Bahrain and Oman, which found that the levels of anxiety in mothers of children with ASD are at 49.2% and 43.8%, respectively [8,9]. Alternatively, a study conducted in China demonstrated the high prevalence of depression and anxiety among mothers of children with ASD at 72.5% and 80.2%, respectively. The study utilized a different tool of assessment. However, its main confounder was socioeconomic status, which is similar to the current study [7].

A retrospective study in Saudi Arabia was conducted using different databases derived across organizations in Riyadh and varying tools of measurement. The authors found that the level of depression was significantly higher among mothers of children with ASD than among mothers of children with typical development. At the same time, the study confirmed a finding similar to the current study, that is, the high levels of stress among mothers of children with ASD, which was considered to be strongly correlated to the development of depressive symptoms [17]. A study conducted in Iran using a small sample size found high levels of anxiety among mothers of children with ASD [18]. Another study conducted in the United States identified the relationship between parenting stress and the behavior of children with ASD. The study found a significant amount of stress on these mothers compared with mothers of children with typical development [19].

Previous studies demonstrated that having larger social networks and support are related to positive well-being among mothers of children with ASD [20]. In Saudi Arabia, a study illustrated that social support exerts a protective effect on the psychological well-being of parents of children with ASD [21]. In the current study, the median scores for stress, anxiety, and depression were inversely related to the median score for the MSPSS. However, this correlation is weak to moderate but statistically significant. Thus, the higher the level of perceived social support among mothers of children with ASD, the better the psychological well-being with respect to stress, anxiety, and depression. In the current study, the mothers of children with ASD perceived above-average social support with an MSPSS median score of 56.6 out of the total score of 84. This result is consistent with those of a study conducted in Turkey, which reported an average score of 51.06 for social support among parents of children with ASD. However, this score is slightly lower than the average score for social support of 69.28 in another study in Turkey. However, the second study was in agreement with the current study, that is, social support is fairly received from family members [22,23].

Limitations and future directions

The design of the current study is cross-sectional in nature, which renders difficult the generalization of the findings or the establishment of causal relationships. Obtaining a large sample within the context of the fragmented care resources for this population is a common challenge in many countries, not only in Saudi Arabia.

To the best of our knowledge, this is the first study in Saudi Arabia to evaluate the correlation between perceived social support and psychological well-being. In this manner, this study is a good start toward further initiatives for the support and better understanding and estimation of the psychological well-being of caregivers of children with ASD in the country.

## Conclusions

The study observed no significant difference in the levels of depression and anxiety between mothers of children with ASD and mothers of children with typical development. High levels of extreme stress were observed among mothers of children with ASD, who reported above-average levels of social support. Thus, the higher the level of perceived social support, the better the psychological well-being of mothers. Nevertheless, a fair proportion of both groups suffer from extremely severe levels of depression and anxiety. Thus, providing mental health services for mothers in need and investigating the underlying factors of extremely severe depression and anxiety are recommended initiatives.
